# Nile Red-Poly(Methyl Methacrylate)/Silica Nanocomposite Particles Increase the Sensitivity of Cervical Cancer Cells to Tamoxifen

**DOI:** 10.3390/polym12071516

**Published:** 2020-07-08

**Authors:** Munther Alomari, Rabindran Jermy Balasamy, Dana Almohazey, Vijaya Ravinayagam, Mohammad Al Hamad, Deena Ababneh, Hiba Bahmdan, Abdul-Hakeem Alomari, Zakaria Mokadem, Abdelhamid Elaissari

**Affiliations:** 1 Department of Stem Cell Biology, Institute for Research and Medical Consultations, Imam Abdulrahman Bin Faisal University, Dammam 31441, Saudi Arabia; daaalmohazey@iau.edu.sa (D.A.); hbahmdan@iau.edu.sa (H.B.); 2 Department of Nano-Medicine Research, Institute for Research and Medical Consultations, Imam Abdulrahman Bin Faisal University, Dammam 31441, Saudi Arabia; rjermy@iau.edu.sa; 3 Deanship of Scientific Research, Department of Nano-Medicine Research, Imam Abdulrahman Bin Faisal University, Dammam 31441, Saudi Arabia; vrnayagam@iau.edu.sa; 4 Department of Pathology, College of Medicine, Imam Abdulrahman Bin Faisal University, Post Box No. 1982, Dammam 31441, Saudi Arabia; mhamad@iau.edu.sa; 5 Department of Basic Sciences and Humanities, College of Engineering, Imam Abdulrahman Bin Faisal University, Post Box No. 1982, Dammam 31441, Saudi Arabia; dababneh@iau.edu.sa; 6 Biomedical Engineering Department, College of Engineering, Imam Abdulrahman Bin Faisal University, Dammam 31441, Saudi Arabia; ahhalomari@iau.edu.sa; 7 Applied Organic Synthesis Laboratory-LSOA, University of Oran Es-Senia, Department of Chemistry, Bp 1524 El M’Naouer, Oran 31000, Algeria; mokadem-zaki@outlook.fr; 8 France Univ Lyon, University Claude Bernard Lyon-1, CNRS, LAGEP-UMR 5007, F-69622 Lyon, France; abdelhamid.elaissari@univ-lyon1.fr

**Keywords:** Nile red (NR), PMMA-NR-Si-TAM, Tamoxifen (TAM), HELA, cancer, delivery

## Abstract

Tamoxifen (TAM) is a hormonal drug and is mainly used as an anti-estrogen in breast cancer patients. TAM binds to estrogen receptors (ERs), resulting in inhibition of estrogen signaling pathways and thus, a downregulation of cell proliferation. Cancer cells with negative or low ER expression will not uptake TAM and will show low response. Poly (methyl methacrylate) (PMMA) nanoparticles were prepared using surfactant-free emulsion polymerization, then were loaded with Nile red (NR), which resulted in PMMA-NR. To enhance TAM delivery to cervical cancer cells (HELA), which is considered ER-negative, we loaded TAM and polymethyl methacrylate nanoparticles-Nile-red into silica (PMMA-NR-Si-TAM). The uptake and intracellular distribution were visualized by confocal laser scanning microscopy, and the in vitro cytotoxic activity was evaluated by MTT (3-(4,5-Dimethylthiazol-2-yl)-2,5-Diphenyltetrazolium Bromide) assay using HELA and non-tumorigenic cell line HFF-1. The sensitivity of HELA (LC50: 207.31 µg/mL) and HFF-1 (LC50: 234.08 µg/mL) to free TAM was very low. However, after the encapsulation of TAM with PMMA-NR, the sensitivity significantly increased HELA (LC50: 71.83 µg/mL) and HFF-1 (LC50: 37.36 µg/mL). This indicates that TAM can be used for the treatment of ER-negative cervical cancer once conjugated to PMMA-NR nanoparticles. In addition, the PMMA-NR formulation appears to be highly suitable for cancer imaging and drug delivery.

## 1. Introduction

In recent years, the insinuation of medicine and nanotechnology (nanomedicine) has broadened the scope for effective and efficient therapeutic approaches on deadly diseases such as cancer, as well as diabetic and other metabolic disorders. In particular, cancer poses a major challenge worldwide. Cancer related deaths are over six million worldwide. Overwhelmingly, new cases and cancer related deaths are expanding each year in all countries. The cancer burden is expected to increase to about 24 million by 2035. Tamoxifen (TAM) is a selective estrogen receptor modulator (SERM) for ER-positive breast cancer patients [1]. TAM blocks estrogen from binding to estrogen receptors α (ERα) and β (ERβ) by competitively binding to ER, thus preventing the transcriptional effects and inhibiting the cell proliferation [2,3]. TAM requires hepatic activation by polymorphic cytochrome CYP2D6 and CY3A4 to form the 4-hydroxytamoxifen and 4-hydroxy-*N*-desmethyltamoxifen (endoxifen). These products have a higher affinity to ER than TAM and thus, a higher ability to inhibit cell proliferation in ER-positive cells [4,5,6,7]. On the other hand, 5%–10% of ER-negative breast cancers displayed anti-tumor activity, suggesting another signaling pathway independent of ER [8]. However, TAM exhibits several dose-dependent drawbacks during clinical administration that lead to blood clotting disorders, vision impairment (retinopathy) and corneal opacities [9]. The therapeutic efficacy of TAM can be improved by using targeted nanocarriers that help to enhance drug permeation [10].

Biocompatible polymeric nanoparticles based on poly(methyl methacrylate), termed as PMMA, have been effective in encapsulating hydrophobic drugs and have inherited several desired characteristics such as, low toxicity, hydrolysis resistant and increased permeability of drugs [11]. The synthesis of such nanoparticles can be accomplished by micro or miniemulsion techniques. For instance, microemulsion preparation of highly pure PMMA has been reported using a low synthesis temperature (less than 35 °C) and UV irradiation technique. Formation of monodispersed PMMA latex particles was observed to have a size distribution average of 9.5 nm [12]. Controlled PMMA nanoparticles (<20 nm) were reported using the differential microemulsion technique. The presence of initiator and synthesis temperature were found to be critical in deciding the particle size, density, molecular weight and polymer chains per particle [13]. On the other hand, the miniemulsion route, which is based on a single step fast polymerization, is also attractive for the synthesis of the photosensitizer zinc (II) phthalocyanine based PMMA [14]. α-terpineol integrated PMMA nanoparticles (up to 400 mg) have been synthesized using the miniemulsion technique. These nanoformulations exhibit high anticancer activity in melanoma cell lines (B16-F10) [15].

However, the use of either microemulsion or miniemulsion techniques is tedious. It often involves the need for a sophisticated high energy experimental setup that is not viable for a commercial scale up process [16]. In addition, the presence of surfactants used in the above-mentioned polymerization processes may affect the cells. Therefore, a simple and effective synthesis technique is needed to develop PMMA based nanoformulations. Dispersion polymerization is also an attractive emulsion technique that has been presented for synthesizing monodispersed PMMA [17]. Recently, we have reported the preparation of monodispersed PMMA by combining the dispersion technique and the surfactant-free emulsion polymerization. The submicron PMMA particle size was shown to be controlled by tuning the methanol/water solvent ratios [18]. Biomedical applications of PMMA have been reported in the field of dental and antibacterial activity [19]. However, the usage of PMMA for targeted biomedical applications involving drug release is hindered due to their reduced mechanical stability, inferior bioactivity and the possibility of causing cell injury [20]. Recently, PMMA composite formation with silica and carbon has been reported for dental and drug delivery [21,22]. In addition, the conjugation of fluorescence to nanoparticles has become an attractive model for tumor imaging. Nile red is a fluorescent stain that is widely used to stain intracellular lipid droplets [23], which makes it an excellent stain for the cell imaging when conjugated to PMMA.

The aim of this study was to develop PMMA-NR-Si-TAM nanocomposite l. This nanoformulation would be able to deliver TAM into cervical cancer cells with a minimal dose while maintaining the therapeutic effect. In addition, it would able to stain the cancer cells with Nile red for evaluating the cell internalization of PMMA/TAM and for cancer cellular imaging.

## 2. Methods

### 2.1. Poly (Methyl Methacrylate) (PMMA)

Poly (methyl methacrylate) (PMMA) latex particles were prepared using 100 g of methyl methacrylate (MMA) and 2 g of potassium persulfate (KPS) in 1 L of deionized water. The polymerization was conducted at 70 °C and under 300 RPM. The hydrodynamic particle size of the final particles was found to be 445 nm, the zeta potential measured at pH 7.5 and in 1 mM NaCl solution was around −63 mV, revealing the high negative character of the particles. This latex was used as a seed for preparing PMMA-NR.

### 2.2. Preparation PMMA-NR Fluorescent Nanoparticle

NR was first solubilized in 3 mL of acetone and was then added directly to 100 mL of seed latex particles (10% wt/vol) under a slow stirrer; the dispersion was incubated for 3 h before acetone evaporation at 40 °C.

### 2.3. Tamoxifen Loading on PMMA-NR/Silica (PMMA-NR-Si)

Firstly, PMMA-NR nanoparticles (86 mg/mL) were dissolved in 50 mL of ethanol for 30 min. Then, 200 mg of pre-dried silica was added and stirred for 1 h. Then, 50 mg of TAM was added and allowed to stir overnight. The solvent was then dried by evaporation at room temperature. The TAM loading over the PMMA-NR-Si nanocomposite was found to be 0.67 mmol·g^−1^ of a nanocarrier.

### 2.4. Tamoxifen Release Study

The TAM release from the PMMA-NR-Si-TAM nanoformulation was studied by the dialysis membrane technique (MWCO = 14,000 Da). Prior to analysis, the activation of the dialysis membrane was carried out by immersing and washing with PBS solution for 1 h. The release ability was studied using 30 mg of nanoformulation/PBS mixture at 37 °C for 307 h. The cumulative percentage at each interval was measured using UV-visible spectroscopy (JASCO). The sample taken for analysis was replaced with a fresh PBS solution.

### 2.5. Material Characterization

The phase characterstics of TAM, PMMA-NR-Si-TAM and silica were measured using bench top X-ray diffraction analysis (Rigaku Multiplex system, Japan). The textural characterization of silica and the PMMA-NR-Si-TAM nanoformulation (surface area and pore size distributions) was measured using Micromeritics, ASAP 2020, USA. The functional interactions of silica, TAM and NR were measured using FT-IR spectroscopy (Perkin Elmer, Waltham, MA, USA). The PMMA-NR, silica and PMMA-NR-Si-TAM sample morphology was analyzed by JEM2100F from JEOL. The sample suspensions for TEM analysis were prepared from the dry sample using ethanol, followed by an ultrasonic treatment for 30 min. A droplet (5 μL) of diluted suspension was deposited into a 300-mesh pure carbon grid and was then kept in the pumping station for 1 h for further drying.

### 2.6. Cell Culture and Treatment

HELA (cervical cancer; ATCC^®^ CCL-2™) and non-cancerous human foreskin fibroblast cells (HFF-1) (ATCC^®^ SCRC-1041™) were used. The cells were grown in a DMEM medium (HyClone, GE Healthcare, Chicago, USA) supplemented with a 10% fetal calf serum (HyClone, GE Healthcare, Chicago, IL, USA), 1% penicillin/streptomycin solution and 1% MEM non-essential amino acids (Thermo Fisher, Waltham, MA, USA) in a 5% CO_2_ incubator (Heracell 150i, Thermo Scientific, Waltham, MA, USA) at 37 °C. The cells were cultured in a 96 well plate (10 × 10^3^ cells/well) (Thermo Fisher, Waltham, MA, USA). At 70% confluent, the cells were treated with 12, 24, 48, 96, 192 and 384 μg/mL concentrations of free TAM and equivalent doses of PMMA-NR-Si-TAM; the calculated concentration of PMMA-NR was also added. The treatment duration was for 24 h, then the relative growth inhibition compared to control cells was estimated using MTT cell survival assay.

### 2.7. Cell Viability Assay

The viability of the cells was measured using a dye reduction test, MTT (3-(4, 5-Dimethylthiazol-2-yl)-2, 5-Diphenyltetrazolium Bromide) assay. A total of 20 µL of MTT (10 mg/mL) was added to each of the drug used and the control cell culture wells of the 96 well plate. The cells were incubated for 3 h at 37 °C, then washed with PBS. A formazan dye that crystalized in live cells was solubilized by 100 µL of isopropanol and 0.04% HCl for 1 h at 37 °C. A multiple reader (Tecan Infinite^®^ 200 PRO, Männedorf, Switzerland) was used to measure the optical density of solubilized dye at 570 nm. The measured optical density (OD) was compared and calculated against control and expressed as percentage of cell survival for each concentration following the equation below:

$$\mathrm{Cell}\;\mathrm{viability}\;\left(\%\right)=\frac{\mathrm{Absorbance}\;\mathrm{of}\;\mathrm{treated}-\mathrm{Absorbance}\;\mathrm{of}\;\mathrm{blank}}{\mathrm{Absorbance}\;\mathrm{of}\;\mathrm{control}-\mathrm{Absorbance}\;\mathrm{of}\;\mathrm{blank}}\times 100$$

### 2.8. Hoechst Staining

The viability of treated and control cells was tested by Hoechst staining. Cells were cultured in an 8 well chamber (30 × 10^3^ cells/well) (Thermo Fisher, Waltham, MA, USA) overnight and were then treated with different concentrations of PMMA-NR and PMMA-NR-Si-TAM for 24 h at 37 °C. The cells were then washed with phosphate buffered saline (PBS) pH 7.4, fixed with ice cold methanol for 5 min and stained with Hoechst (Sigma Aldrich, St. Louis, MO, USA) for 10 min in the dark. Cells were washed again with PBS, dried up at room temperature and mounted with a ProLong^®^ Gold Antifade reagent (Thermo Fisher, Waltham, MA, USA). Images were obtained using a confocal microscopy and were developed by ZEN software (Zeiss, Oberkochen, Germany).

### 2.9. Statistical Analysis

The MTT cell viability assay results were subjected to the one-way ANOVA, followed by Dunnett’s post hoc test of GraphPad Prism software (GraphPad, La Jolla, CA, USA) on three independent sets of experiments conducted in triplicates. *p* values <0.05 were considered significant.

## 3. Results and Discussion

### 3.1. PMMA-NR Properties

**Figure 1:**
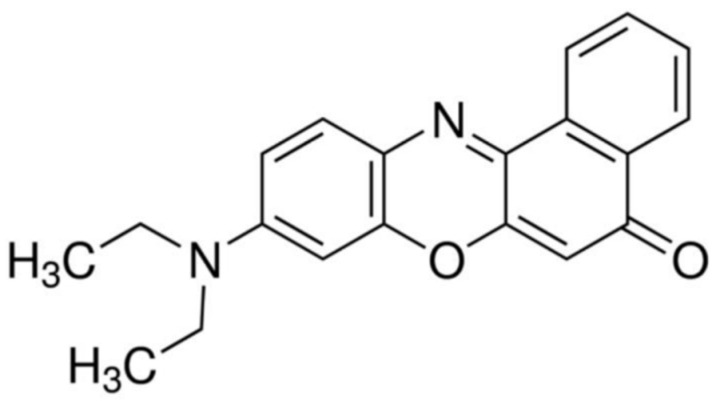
Chemical structures of PMMA-NR.

The hydrodynamic size of the PMMA-NR (Figure 1) measured in 1 mM NaCl was found to be 439 nm and the zeta potential measured at pH 7.5; 1 mM NaCl was found to be −62 mV.

**Figure 2:**
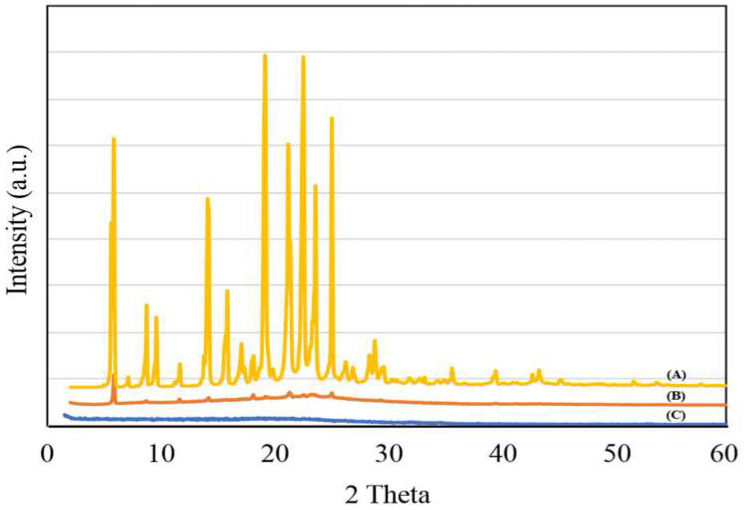
The XRD spectra for (**A**) free drug TAM, (**B**) PMMA-NR-Si-TAM and (**C**) silica.

Figure 2A–C shows the XRD pattern of TAM, PMMA-NR-Si-TAM and silica. TAM exhibited several characteristics, such as crystalline peaks at the 2 theta range between 5–50° (Figure 2A). PMMA is reported to exhibit an amorphous (2θ = 9.16°) and crystalline peak (2θ = 19.12°) [24]. In the present case, PMMA-NR-Si-TAM showed the disappearance of major crystalline peaks, which confirms the amorphous transformation of the TAM drug inside the pores of silica (Figure 2B). In particular, the presence of small peaks clearly reflected similar amorphous characteristics of silica (Figure 2C). Such drastic peak transitions of TAM and PMMA clearly reciprocate the critical role of spherical silica in improving solubility, bioavailability and pharmacological applications [25].

**Figure 3:**
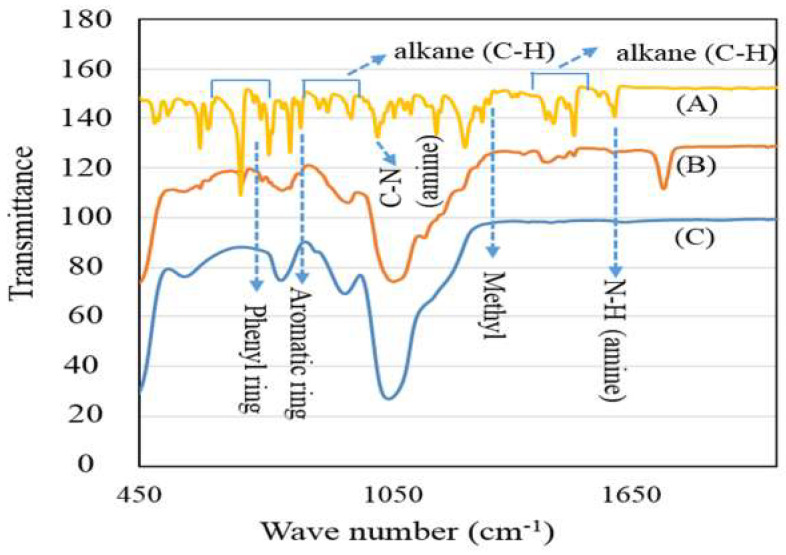
The functional group interactions of drug TAM (**A**), (**B**) PMMA-NR-Si-TAM and (**C**) silica, which were studied using FT-IR.

Figure 3 shows the interaction of TAM in PMMA-NR-Si-TAM using FT-IR spectroscopy. TAM showed distinct bands due to amine (1600 cm^−1^), aromatic ring substitution (700–764 cm^−1^), alkane C-H group (890–960 cm^−1^ and 1440–1510 cm^−1^), amine C-N band (1030–1050 cm^−1^), methyl (1305 cm^−1^) and amine N-H band (1610 cm^−1^) (Figure 3A) [26]. The phase analysis using XRD indicated the presence of amorphous TAM over silica (Figure 2). Altmeyer et al. (2006) indicated that such a physical state transformation of the drug mainly occurs due to the favorable entrapment ability between nanocarrier matrices and TAM [27]. In the case of PMMA-NR-Si-TAM, the presence of broad and lower intensity of TAM functional groups indicates the presence of the interactive drugs in the amorphous state over silica support (Figure 3B).

**Figure 4:**
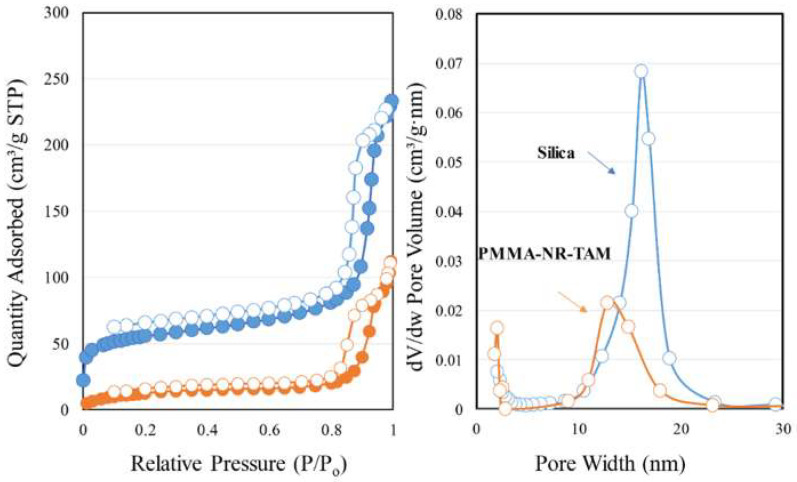
The surface area and pore size distributions of silica and PMMA-NR-Si-TAM.

**Figure 5:**
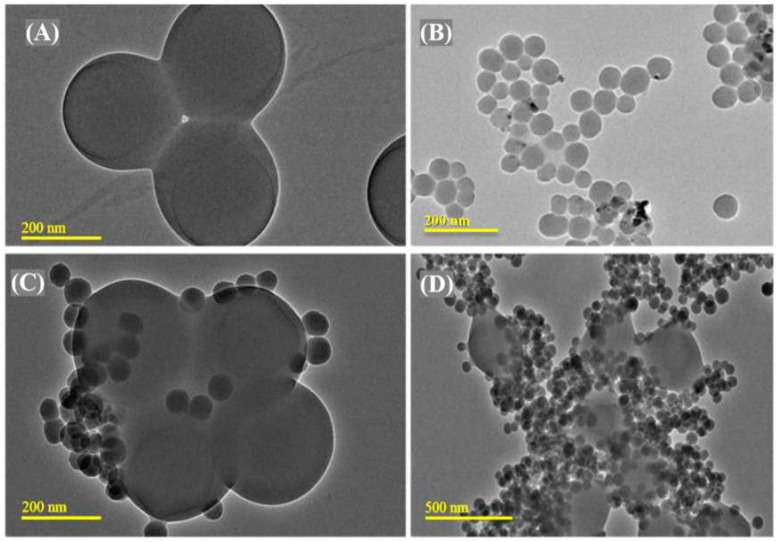
TEM analysis of (**A**) PMMA-NR, (**B**) silica and (**C**,**D**) PMMA-NR-Si-TAM at 200 nm and 500 nm.

The textural properties (surface area and pore size distributions) of silica and PMMA-NR-Si-TAM were measured using nitrogen adsorption isotherm. Silica showed the surface area of 178 m^2^/g. The coaddition of NR/PMMA and TAM reduced the surface area to 42 m^2^/g (Figure 4). The percentage decrease of the surface area was found to be about 76%, while pore size distribution measurements showed a decrease of about 54% from 0.35 cm^3^/g to 0.16 cm^3^/g (Figure 4). This trend clearly indicates an effective surface functionalization and pore filling of nanosized TAM and NR inside the texture of silica. The morphology pattern of the PMMA-NR, silica and PMMA/TAM/silica nanocomposite was analyzed using transmission electron microscopy (Figure 5). PMMA can be synthesized with a diameter ranging between 100–300 nm. In our present case, PMMA-NR exhibited a spherical morphology with a particle size of about 270 nm (Figure 5A). Silica showed uniform spherical nanospheres with a particle size of about 80 nm (Figure 5B). The PMMA-NR-Si-TAM nanocomposite at different scale bars of 200 nm and 500 nm showed an excellent composite formation where the silica functionalized with TAM tended to bound on the surface of PMMA-NR.

### 3.2. In Vitro Tamoxifen Release Study

Table 1 and Supplementary Figure S1 present the drug release profile of PMMA-NR-Si-TAM. The diffusion of TAM from nanocarrier was found to occur in a controlled fashion. The cumulative percentage of TAM released was about 2% in 24 h. Later, the drug delivery increased and drug release steadied at about 30% in the period of 12 days. In the case of phospholipid/polymer micelles, a release of about 75% was observed within 24 h [28]. TAM over lipid carrier showed a fast TAM release that reached up to 90% within 12 h [29]. The controlled drug release of TAM for longer durations showed an enhanced solubilization of TAM and its effective diffusion into pores of the PMMA-NR-Si composite.

### 3.3. In Vitro Anti-Cancer Studies

**Figure 6:**
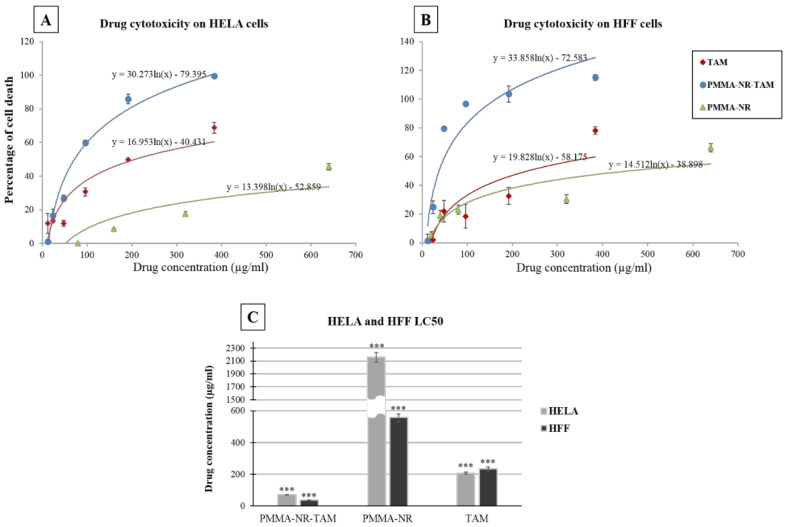
Dose response curves showing the cytotoxic activities of PMMA-NR-Si-TAM, PMMA-NR and free TAM on HELA and HFF-1 cells. (**A**) Logarithmic dose response curve of the HELA cervical cell line. (**B**) Logarithmic dose response curve of normal cell lines HFF-1. (**C**) LC50 of PMMA-NR-Si-TAM, PMMA-NR and free TAM on HELA and HFF-1 cells. The results are presented as average ± standard deviation of three independent measurements (*** *p* < 0.001, n = 3).

The TAM, PMMA-NR and PMMA-NR-Si-TAM cytotoxicities were tested on cancerous and normal cell lines. The resulted data were plotted as a logarithmic curve in excel (Figure 6A,B) in order to get the LC50 of nanoparticles and free drug using the logarithmic trendline. The *p* values and standard deviations of three replicates were measured. The decrease in cell proliferation was observed with an increased concentration of PMMA-NR-Si-TAM to show complete cell death at the concentration of 384 µg/mL in HELA cells (Figure 6A) and around 96 µg/mL in HFF-1 (Figure 6B). At the same concentration of free TAM, the percentage of cell death was 68% and 78% in HELA and HFF-1, respectively. While at 320 µg/mL of PMMA-NR, cell death was only at 17.5% and 30% in HELA and HFF-1, respectively (Figure 6A,B). The LC50 of the logarithmic trendline equation was calculated. The average of three replicates was plotted with standard deviation (Figure 6A) and the *p* values were calculated using a two-tailed test (Table 2). The LC50 of PMMA-NR-Si-TAM for HELA cells was 71.83 µg/mL and 37.36 µg/mL for HFF-1 cells, indicating that HFF-1 is more responsive to PMMA-NR-Si-TAM than HELA cells. The cumulative percentage of TAM using dialysis membrane in pH 7 for 24 h was 20 µg (Table 1). On the other hand, the LC50 of PMMA-NR-Si-TAM for non-cancerous HFF-1 cells (pH 7.4) was 37.36 µg/mL, while for HELA cells it was 71.83 µg/mL (acidic pH below 6). The study showed that at normal pH, less TAM was released, while at tumour acidic pH condition, TAM release was increased. In addition, these data indicate that PMMA-NR-TAM is not selective towards cancer cells.

In addition, HFF-1 showed a higher sensitivity toward PMMA-NR (LC50: 556.89 µg/mL) compared to HELA (LC50: 2158.55 µg/mL). On the other hand, the TAM cytotoxicity was higher in HELA cells (LC50: 207.31 µg/mL) than in HFF-1 (LC50: 234.08 µg/mL). The results revealed the high efficacy of the PMMA-NR, silica and TAM combination in the inhibition of cell proliferation in comparison to PMMA-NR and free TAM alone.

**Figure 7:**
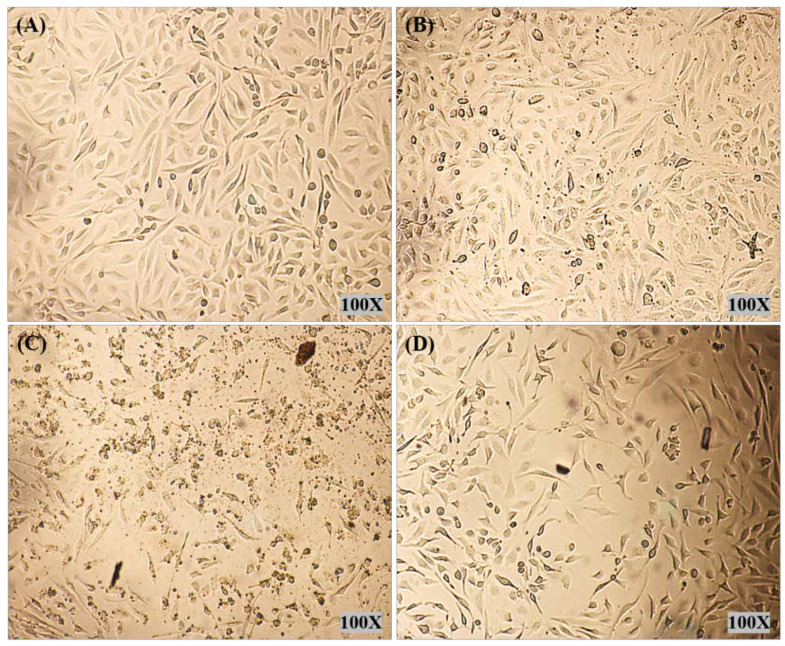
Cell morphology images of HELA cells after 24 h of treatment. HELA cells were treated at a concentration of 71.83 µg/mL with (**A**) control (no treatment), (**B**) PMMA-NR, (**C**) PMMA-NR-Si-TAM and (**D**) free TAM. The small black dots are the nanoparticles and the brown debris is the dead cells.

**Figure 8:**
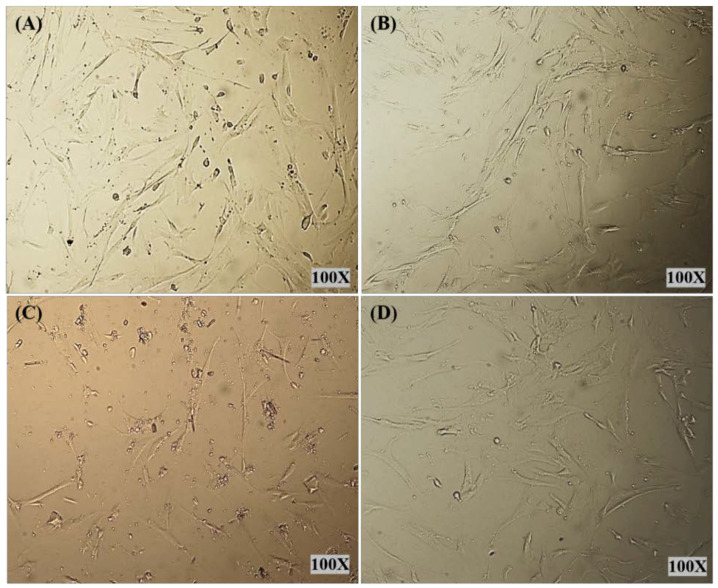
Cell morphology images of HFF-1 cells after 24 h of treatment. HFF-1 cells were treated at a concentration of 37.36 µg/mL with (**A**) control, (**B**) PMMA-NR, (**C**) PMMA-NR-Si-TAM and (**D**) free TAM.

Light microscopy images were recorded for treated HELA and HFF-1 with (A) control, (B) PMMA-NR, (C) PMMA-NR-Si-TAM and (D) TAM (Figure 7 and Figure 8). In the case of HELA, cells were treated with a concentration of 71.83 µg/mL of PMMA-NR-Si-TAM, which is the LC50 in HELA (Figure 7C, Table 2). While in HFF-1, cells were treated with 37.36 µg/mL of PMMA-NR-Si-TAM, which is the LC50 concentration in HFF-1 (Figure 8C, Table 2). The treatment using the LC50 concentration aimed to show the visual effect of PMMA-NR-Si-TAM compared with the PMMA-NR and free TAM treatments on cell proliferation (Figure 7 and Figure 8). Nanoparticles and the drug combination (PMMA-NR-Si-TAM) showed the highest cytotoxicity in HELA cells with a 50%–60% reduction in cell numbers and a noticeable death (brown debris) (Figure 7C) in comparison to control. The cell proliferation in HELA cells treated with PMMA-NR at 71.83 µg/mL was similar to the control, as it did not show a noticeable cell number reduction or death. The black dots are the PMMA-NR nanoparticles. On the other hand, the TAM treatment with the same concentration (71.83 µg/mL) showed about 25% reduction in cell numbers (Figure 7D). HFF-1 cells with free TAM and PMMA-NR treatments (37.36 µg/mL) showed only about 17% and 15% reduction in cell numbers in comparison to the control (Figure 8B,D). While in PMMA-NR-Si-TAM treatment, there was more than a 50% decrease in cell numbers (Figure 8C) compared to the control (Figure 8C). In addition, PMMA-NR-Si-TAM treatment changed the morphology of the cells in HFF-1. The cellular side extensions were highly reduced and the cells became thinner, which resulted in separating the cells from each other (Figure 8C).

**Figure 9:**
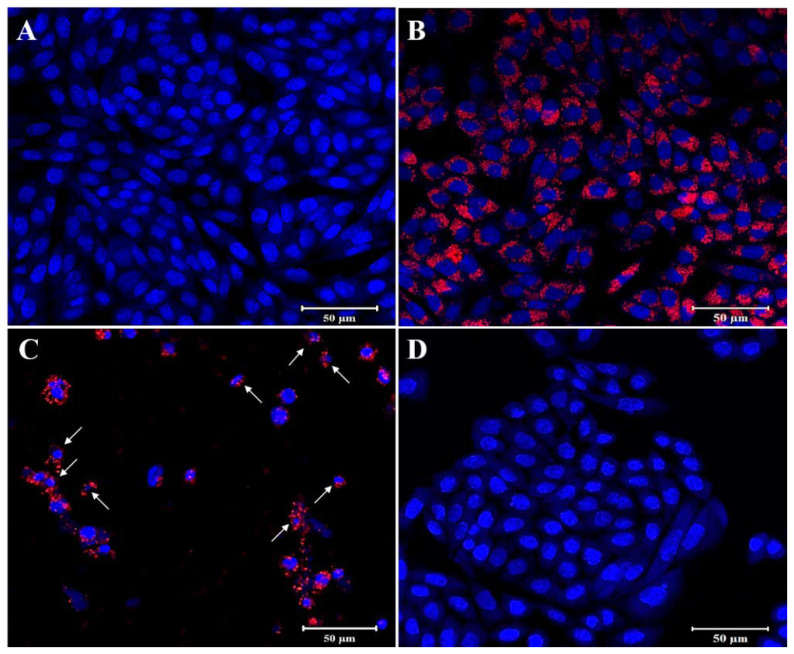
Confocal laser scanning microscopy images of HELA cells after 24 h of treatment. In the images, NR is shown in red color and the nucleus is stained with Hoechst in blue. (**A**) Control (no treatment), (**B**) PMMA-NR treatment (71.83 µg/mL), (**C**) PMMA-NR-Si-TAM treatment (71.83 µg/mL), (**D**) TAM treatment (71.83 µg/mL). The arrows indicate the nucleus structural changes. White arrow: DNA condensation. These images clearly showed efficient uptake of PMMA-NR and PMMA-NR-Si-TAM by the HELA cells. Scale bar: 50 μm.

**Figure 10:**
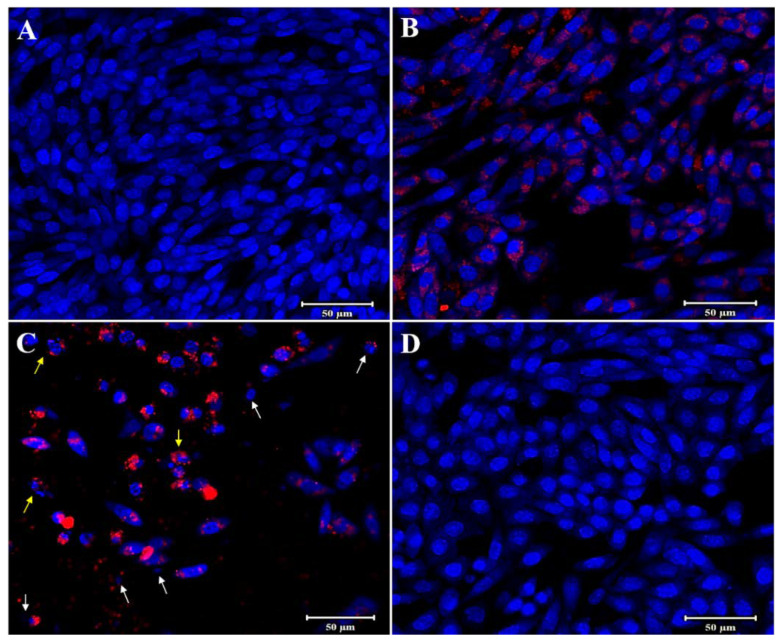
Confocal laser scanning microscopy images of HFF-1 cells after 24 h of treatment. In the images, NR is shown in red color and the nucleus is stained with Hoechst in blue. (**A**) Control (no treatment), (**B**) PMMA-NR treatment (37.36 µg/mL), (**C**) PMMA-NR-Si-TAM treatment (37.36 µg/mL), (**D**) TAM treatment (37.36 µg/mL). The arrows indicate the nucleus structural changes. White arrow: DNA condensation, yellow arrow: nuclear fragmentation/apoptotic bodies. These images clearly showed efficient uptake of PMMA-NR and PMMA-NR-Si-TAM by the HFF-1 cells. Scale bar: 50 μm.

The fluorescent images presented of HELA and HFF-1 cells further illustrated the fluorescent labeling ability of PMMA-NR and the effects of PMMA-NR-Si-TAM on cellular proliferation (Figure 9 and Figure 10). PMMA-NR treated cells (Figure 9B and Figure 10B) showed a red fluorescent color all around the cell, with very low cellular cytotoxicity (LC50: HELA (2158.55 µg/mL), HFF (556.89 µg/mL)). This indicates that the PMMA-NR may be used for cancer imaging. The encapsulation of TAM in PMMA-NR facilitated the entry of TAM into the cells. Compared to PMMA-NR and free TAM, treatment with PMMA-NR-TAM resulted in a high cellular cytotoxicity, cell number reduction, DNA condensation, nuclear fragmentation and apoptotic bodies (Figure 9C and Figure 10C). The cellular uptake and intracellular distribution of PMMA-NR and PMMA-NR-Si-TAM were studied using confocal laser scanning microscopy. Cells incubated with PMMA-NR showed cytosolic NR staining (Figure 9B and Figure 10B), while cells incubated with PMMA-NR-Si-TAM showed cytosolic and nuclear NR staining. This indicated the ability of PMMA-NR-Si-TAM of intracellular distribution in the cytoplasm and nucleus.

## 4. Conclusions

In this study we successfully labeled PMMA with NR and TAM, which resulted in a compact particle. The coaddition of PMMA-NR and TAM showed an effective surface functionalization and pore filling of nanosized TAM and NR inside the texture of silica. The cumulative percentage of TAM release was about 2% in 24 h. In addition, the controlled drug release of TAM for longer durations showed an enhanced solubilization of TAM and its effective diffusion into the pores of the PMMA-NR-Si composite.

PMMA-NR-Si-TAM was able to deliver TAM into the cells and attenuated the cervical cancer cells compared to PMMA-NR or free TAM treatment. PMMA-NR-Si-TAM treatment resulted in cell debris, DNA condensation and nuclear fragmentation in the HELA cancer cell line, as well as NR labeling for viable cells. Furthermore, LC50 of PMMA-NR-Si-TAM was very low in comparison to that without TAM. This indicates that TAM can be used for the treatment of cervical cancer cells at a low dose once conjugated to PMMA-NR-Si nanoparticles while maintaining the therapeutic effect. In addition, the PMMA-NR formulation is highly suitable for cancer imaging and drug delivery.

## Figures and Tables

**Table 1:** In vitro TAM drug release data.

Time (h)	Drug Release (mg)	Cumulative Drug Release (%)
1	0.004	0.48
6	0.012	1.27
12	0.014	1.64
24	0.020	2.03
48	0.034	3.55
72	0.137	13.98
120	0.204	21.26
240	0.234	26.01
300	0.263	29.94

**Table 2:** HELA and HFF-1 treated with PMMA-NR-Si-TAM, PMMA-NR and free TAM.

Drug	HFF			HELA		
	LC50 (µg/mL)	SD	*p* Value	LC50 (µg/mL)	SD	*p* Value
PMMA-NR-Si-TAM	37.36	2.72	3.33 × 10^−6^	71.83	0.88	1.09 × 10^−6^
PMMA-NR	556.89	24.32	3.33 × 10^−6^	2158.55	73.24	1.09 × 10^−6^
TAM	234.08	11.90	9.72 × 10^−6^	207.31	7.89	7.4 × 10^−6^

## Supplementary Materials

The following are available online at https://www.mdpi.com/2073-4360/12/7/1516/s1, Figure S1: Tamoxifen release profile from PMMA-NR-Si-TAM.

## References

[1] Goetz M.P., Knox S.K., Suman V.J., Rae J.M., Safgren S.L., Ames M.M., Visscher D.W., Reynolds C., Couch F.J., Lingle W.L. (2006). The impact of cytochrome P450 2D6 metabolism in women receiving adjuvant tamoxifen. Breast Cancer Res. Treat..

[2] Goldstein S.R., Siddhanti S., Ciaccia A.V., Plouffe L. (2000). A pharmacological review of selective oestrogen receptor modulators. Hum. Reprod. Update.

[3] Sato M., Glasebrook A.L., Bryant H.U. (1994). Raloxifene: A selective estrogen receptor modulator. J. Bone Miner. Metab..

[4] Mürdter T.E., Kerb R., Turpeinen M., Schroth W., Ganchev B., Böhmer G.M., Igel S., Schaeffeler E., Zanger U., Brauch H. (2011). Genetic polymorphism of cytochrome P450 2D6 determines oestrogen receptor activity of the major infertility drug clomiphene via its active metabolites. Hum. Mol. Genet..

[5] Johnson A.C., Jürgens M.D., Williams R., Kümmerer K., Kortenkamp A., Sumpter J.P. (2008). Do cytotoxic chemotherapy drugs discharged into rivers pose a risk to the environment and human health? An overview and UK case study. J. Hydrol..

[6] Brauch H., Mürdter T.E., Eichelbaum M., Schwab M. (2009). Pharmacogenomics of Tamoxifen Therapy. Clin. Chem..

[7] Kisanga E.R., Mellgren G., Lien E.A. (2005). Excretion of hydroxylated metabolites of tamoxifen in human bile and urine. Anticancer Res..

[8] Manna S., Holz M.K. (2016). Tamoxifen Action in ER-Negative Breast Cancer. Signal Transduct. Insights.

[9] Majd M.H., Asgari D., Barar J., Valizadeh H., Kafil V., Abadpour A., Moumivand E., Mojarrad J.S., Rashidic M.-R., Coukos G. (2013). Tamoxifen loaded folic acid armed PEGylated magnetic nanoparticles for targeted imaging and therapy of cancer. Colloids Surf. B Biointerfaces.

[10] Boyd B.J., Bergström C.A., Vinarov Z., Kuentz M., Brouwers J., Augustijns P., Brandl M., Bernkop-Schnürch A., Shrestha N., Preat V. (2019). Successful oral delivery of poorly water-soluble drugs both depends on the intraluminal behavior of drugs and of appropriate advanced drug delivery systems. Eur. J. Pharm. Sci..

[11] Sahu A., Solanki P., Mitra S. (2018). Curcuminoid-loaded poly(methyl methacrylate) nanoparticles for cancer therapy. Int. J. Nanomed..

[12] Xie L., Li Z., Li X. (2019). Preparation of PMMA nanolatex via microemulsion polymerization initiated by UV direct radiation at low temperature. Ferroelectrics.

[13] Yuan L., Wang Y., Pan M., Rempel G.L., Pan Q. (2013). Synthesis of poly(methyl methacrylate) nanoparticles via differential microemulsion polymerization. Eur. Polym. J..

[14] Feuser P.E., Gaspar P.C., Jacques A.V., Tedesco A.C., Silva M.C.D.S., Ricci-Júnior E., Sayer C., De Araújo P.H.H. (2016). Synthesis of ZnPc loaded poly(methyl methacrylate) nanoparticles via miniemulsion polymerization for photodynamic therapy in leukemic cells. Mater. Sci. Eng. C.

[15] Batista F.A., Fontele S.B.C., Santos L.K.B., Filgueiras L.A., Nascimento S.Q., Sousa J.M.D.C.E., Gonçalves J.C.R., Mendes A.N. (2020). Synthesis, characterization of α-terpineol-loaded PMMA nanoparticles as proposed of therapy for melanoma. Mater. Today Commun..

[16] Bang J., Rhee S.E., Kim K., Lee B.H., Choe S. (2012). Effect of hydrogen peroxide on the UCST behavior of PMMA in the modified dispersion polymerization. Macromol. Res..

[17] Peng B., Van Der Wee E., Velikov K.P., Van Blaaderen A. (2012). Synthesis of Monodisperse, Highly Cross-Linked, Fluorescent PMMA Particles by Dispersion Polymerization. Langmuir.

[18] Badr I.H.A., Lahmar H., Kaewsaneha C., Saïdi-Besbes S., Elaïssari A. (2018). Preparation and Characterization of Poly(methyl methacrylate) Particles by Combined Dispersion and Emulsion Polymerization. Macromol. Res..

[19] Gad M.M., Fouda S.M., Al-Harbi F.A., Näpänkangas R., Raustia A. (2017). PMMA denture base material enhancement: A review of fiber, filler, and nanofiller addition. Int. J. Nanomed..

[20] De Mori A., Di Gregorio E., Kao A.P., Tozzi G., Barbu E., Sanghani-Kerai A., Draheim R.R., Roldo M. (2019). Antibacterial PMMA Composite Cements with Tunable Thermal and Mechanical Properties. ACS Omega.

[21] Shanmugasundar S., Kannan N., Sundaravadivel E., Zsolt S., Mukunthan K.S., Manokaran J., Narendranath J., Kamalakannan V.P., Kavitha P., Prabhu V. (2019). Study on the inflammatory response of PMMA/polystyrene/silica nanocomposite membranes for drug delivery and dental applications. PLoS ONE.

[22] Liang T., Yan C., Zhou S., Zhang Y., Yang B. (2017). Carbon black reinforced polymethyl methacrylate (PMMA)-based composite particles: Preparation, characterization, and application. J. Geophys. Eng..

[23] Greenspan P., Mayer E.P., Fowler S.D. (1985). Nile red: A selective fluorescent stain for intracellular lipid droplets. J. Cell Biol..

[24] Abasi C.Y., Wankasi D., Dikio C. (2018). Adsorption Study of Lead(II) Ions on Poly(methyl methacrylate) Waste Material. Asian J. Chem..

[25] Liu N., Couto R., Seifried B., Moquin P., Delgado L., Temelli F. (2018). Characterization of oat beta-glucan and coenzyme Q10-loaded beta-glucan powders generated by the pressurized gas-expanded liquid (PGX) technology. Food Res. Int..

[26] Mukherjee B., Maji R., Dey N.S., Satapathy B.S., Mondal S. (2014). Preparation and characterization of Tamoxifen citrate loaded nanoparticles for breast cancer therapy. Int. J. Nanomed..

[27] Altmeyer C., Karam T.K., Khalil N.M., Mainardes R.M. (2016). Tamoxifen-loaded poly(L-lactide) nanoparticles: Development, characterization and in vitro evaluation of cytotoxicity. Mater. Sci. Eng. C.

[28] Kumar P., Kumar R., Usha K., Malik R., Sharma G., Chitkara D., Katare O., Raza K. (2016). Biocompatible Phospholipid-Based Mixed Micelles for Tamoxifen Delivery: Promising Evidences from in vitro Anticancer Activity and Dermatokinetic Studies. AAPS PharmSciTech.

[29] How C.W., Rasedee A., Manickam S., Rosli R. (2013). Tamoxifen-loaded nanostructured lipid carrier as a drug delivery system: Characterization, stability assessment and cytotoxicity. Colloids Surf. B Biointerfaces.

