# Supplementary figures and images for: Nile Red-Poly(Methyl Methacrylate)/Silica Nanocomposite Particles Increase the Sensitivity of Cervical Cancer Cells to Tamoxifen
Source: Polymers (Basel). 2020 Jul 8;12(7):1516. doi: 10.3390/polym12071516 (PMC7408027; doi:10.3390/polym12071516)

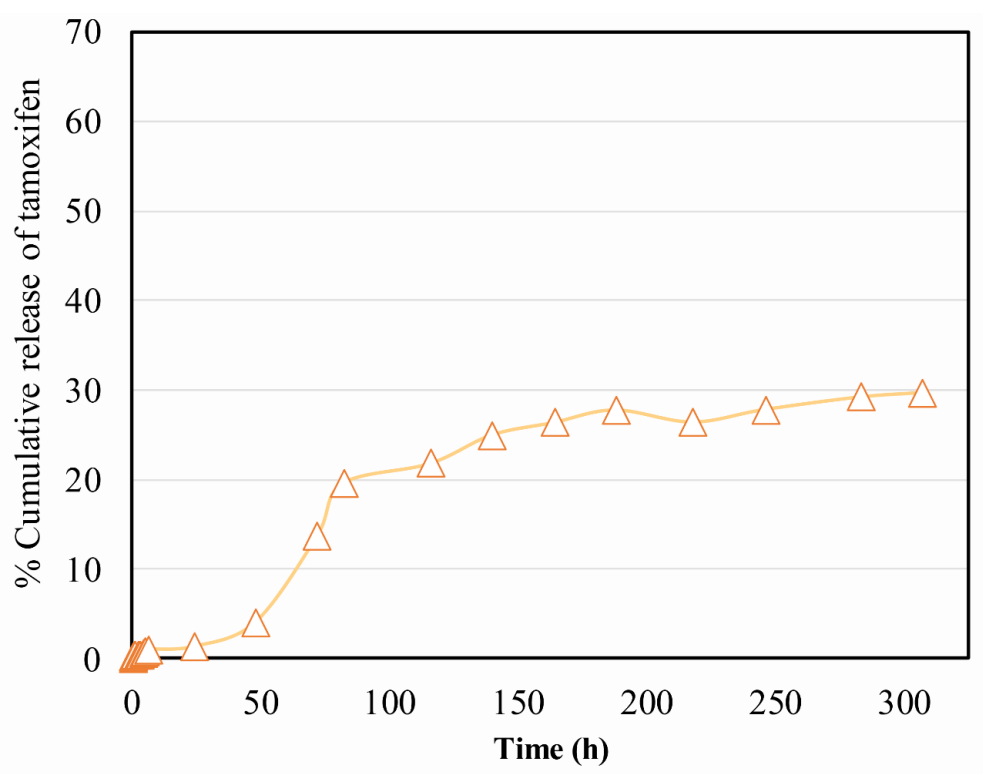

**Figure S1.** Tamoxifen release profile from PMMA-NR-Si-TAM.

Supplement: Supplementary file 1 [file polymers-12-01516-s001.pdf]
